# Optimising personal continuity in general practice: process evaluation of a toolkit

**DOI:** 10.3399/BJGP.2025.0401

**Published:** 2026-06-16

**Authors:** Lex JJ Groot, Annemarie A Uijen, Henk J Schers, Jako S Burgers, Martin Smalbrugge, Henriëtte E van der Horst, Otto R Maarsingh

**Affiliations:** 1 Department of General Practice, Amsterdam UMC, Vrije Universiteit Amsterdam, Amsterdam; Aging and Later Life, Amsterdam Public Health, Amsterdam, Netherlands; 2 Department of Primary and Community Care, Radboud University Nijmegen Medical Centre, Nijmegen, Netherlands; 3 Maastricht University, Department of General Practice, Care and Public Health Research Institute (CAPHRI), Maastricht, Netherlands; 4 Department of Medicine for Older People, Amsterdam UMC, Vrije Universiteit Amsterdam, Amsterdam; Aging and Later Life, Amsterdam Public Health Research Institute, Amsterdam, Netherlands

**Keywords:** general practice, continuity of care, implementation science, physician–patient relations

## Abstract

**Background:**

Personal continuity — having a GP who knows and follows the patient — is a core value of general practice, yet changes in society and health care have challenged its provision. Therefore, we developed TOOL-kit: an intervention to opTimise persOnal cOntinuity for oLder patients in general practice, a multi-component intervention aimed to optimise personal continuity for patients aged ≥65 years.

**Aim:**

To evaluate TOOL-kit’s implementation and identify barriers and facilitators influencing its delivery.

**Design and setting:**

This mixed-methods study was embedded in the main effect trial (August 2020 to April 2022).

**Method:**

Implementation was assessed via 3-monthly surveys and 93 interviews with GPs and practice staff (*n* = 221) from 31 general practices. Based on Saunders’ paradigm, it was evaluated on the process indicators of recruitment and reach, fidelity (adherence to protocol), dose delivered (extent of improvement plans), and dose received (goal achievement, sustained organisational change). Barriers and facilitators were evaluated on the intervention, staff, organisational, and sociopolitical levels.

**Results:**

Of 31 practices, 23 adhered to protocol. TOOL-kit was delivered at a (very) low dose in 17 of 31 practices. At study end, 55% of practice goals were met. After 24 months, 19 of 31 practices had observed sustained positive effects regarding personal continuity. A good-quality improvement infrastructure facilitated TOOL-kit implementation, while work pressure and staff shortages, exacerbated by COVID-19, were seen as the main barriers for implementation.

**Conclusion:**

Despite implementation challenges, TOOL-kit was able to enhance work procedures and improve the focus on personal continuity within practice. Fostering practices’ quality improvement infrastructure and decision-making processes could improve implementation.

## How this fits in

It is increasingly difficult for GPs to maintain personal continuity — having a GP who knows and follows the patient — owing to changes in society and healthcare systems. Yet interventions aimed at improving personal continuity are often not formally evaluated. This study contributes by examining the implementation of an adaptable, multicomponent intervention designed to support general practices in optimising personal continuity: TOOL-kit. Our findings suggest that a multimodal approach may be effective when GPs are provided with adequate resources (such as time and personnel), alongside structured decision-making, a culture of quality improvement, and team stability.

## Introduction

Personal continuity is defined as having a personal GP who knows the patients and with whom the patient can develop an ongoing relationship.^1,2^ Personal continuity is associated with many benefits, including higher levels of patient and GP satisfaction,^3–6^ better patient–doctor relationship,^3,5^ better patient quality of life,^7–9^ higher medication adherence,^10–12^ lower use of hospital services,^4,12–17^ increased physician productivity,^18^ reduced workload within practice and hospital, and an overall reduction in healthcare costs.^19–22^ Additionally, higher levels of personal continuity are associated with a lower mortality rate.^20,21,23–28^

Over time, changes in society and health care have challenged the provision of personal continuity.^29,30^ GPs increasingly work part time, organise themselves into larger group practices, and are less likely to be connected to a single practice for a long period of time, making it more difficult to build and maintain a personal relationship with their patients.^31–33^ Certainly, in an ageing population, there is an increasing number of older patients (aged ≥65 years) with chronic diseases who are at higher risk of fragmentation of care because they require more and multiple healthcare providers in different settings.^34^ At the same time, these patients may benefit more from personal continuity than younger, more healthy people.^12,16,35–38^

To support GPs in responding and adapting to ongoing changes in society and health care, we developed TOOL-kit: an intervention to opTimise persOnal cOntinuity for oLder patients in general practice.^39^ TOOL-kit was developed with input from GPs, patients, practice assistants, and nurses using the Medical Research Council (MRC) framework for developing complex interventions.^39–42^ TOOL-kit was first evaluated in a small-scale pilot where two GPs reviewed it and were asked to evaluate TOOL-kit on content, feasibility, and validity in a semi-structured interview. In another study, we evaluated the effectiveness of TOOL-kit on personal continuity experienced by older patients in a cluster randomised pragmatic trial. We did not find a significant effect, despite the intervention being perceived as feasible and acceptable by practice employees.^43^

Although many interventions for personal continuity exist, these interventions are often not systematically evaluated, leading to doubts about their efficacy and whether they should be implemented outside of their context.^44^ Exploring why a well-received intervention did not yield measurable effects may provide valuable insights into general practices’ approaches to quality improvement and continuity of care, informing future efforts to optimise personal continuity for older patients.

In this study, we report on the evaluation of the process of applying TOOL-kit in general practices and to review the context and delivery to identify barriers and facilitators for its implementation.

## Method

### Design

This process evaluation used qualitative and quantitative methods to collect data on TOOL-kit application. Data were assessed using 3-monthly surveys and interviews with GPs and practice staff after the intervention started. Process indicators were used to analyse implementation, considering a list of relevant barriers and facilitators.

We used the SQUIRE guidance for the reporting of this process evaluation.^45^

### Setting

The present study was embedded in the main trial - that is, a cluster-randomised, stepped-wedge, pragmatic trial. The study protocol and results of the effectiveness study were published elsewhere.^39,43^ In summary, we included 139 GPs, 64 practice assistants, 12 nurses, and six other practice staff (*n* = 221 staff in total) from 32 general practices with three or more regular GPs. In each practice, a coordinating GP was commissioned as project leader and contact person for the researchers. The intervention, TOOL-kit, was introduced in three wedges: wedge 1, directly after baseline measurements; wedge 2, after 3 months of usual care; and wedge 3, after 6 months of usual care. The primary outcome was continuity of care at patient level measured with a validated patient questionnaire (Nijmegen Continuity Questionnaire). Secondary outcomes included work stress and satisfaction and perceived level of personal continuity measured among practice employees.

#### Description of Dutch general practice

Almost all Dutch general practices are owned and managed by self-employed GPs and are not part of a larger organisation or regulated by the government. Most practices have a part-time practice manager supporting the GP(s) in management.

All practices employ practice assistants and nurses. The practice assistant is the first point of contact for patients’ telephone triage and planning consultations. They also may perform supportive medical tasks (for example, blood pressure measurement, urine testing, removing sutures). Practice nurses support the GP in the care of patients with chronic conditions, such as diabetes mellitus, asthma and chronic obstructive pulmonary disease (COPD), hypertension, and cardiovascular disease.^45^ As GP practices are mostly self-owned and self-led, the exact organisation, responsibilities and tasks are flexible and may vary between practices of different sizes and structures.

GPs are paid by a mix of capitation fee, fee for services (for example, consultations, visits, minor surgery), and a contract with the health insurer for chronic care.

A long-running, well-known, national quality improvement incentive programme is the national practice accreditation, organised by the Dutch College of General Practitioners. With the accreditation, practices are provided guidance and are stimulated to formulate and execute a quality improvement plan adhering to Specific, Measurable, Attainable, Realistic/Relevant, and Time-bound (SMART) guidelines and organise their quality improvement in a plan-do-check-act (PDCA) cycle in exchange for a quality mark.^46,47^

The trial took place during the COVID-19 pandemic. In this setting, general practices had to deal with major changes in the practice organisation processes because of increased staff shortages owing to COVID-19 infections or quarantine, increased workload owing to care for patients with COVID-19, vaccinations, and changing COVID-19 prevention legislation.^48,49^

### Description of the intervention

TOOL-kit is a multi-component intervention intended to evaluate and improve personal continuity in general practice. The development of and TOOL-kit itself are included in our main trial publication.^43^ In summary, the main components of TOOL-kit consist of 34 adaptable strategies, integrated in a digital tool. These strategies entail suggestions on what a practice could do to improve personal continuity (for example, instruct practice assistants to schedule appointments with the patient’s regular GP, use electronic health records [EHR] search strategies to identify patients with low continuity of care, or instruct locum GPs to write a handover report), accompanied by guidance to implement a suggestion into daily clinical practice. The TOOL-kit guidance involves four steps (Box 1). TOOL-kit was designed for potential integration in the national practice accreditation.

Box 1. **Steps of the TOOL-kit protocol**

**Step 1:** Run practice scan — a short survey with yes or no questions on current activities to identify possibilities for improving personal continuity

**Step 2:** Select strategies for improvement — using practice scan results, a list of recommended improvement strategies is generated by TOOL-kit. Practices may select any number of recommended strategies from this list

**Step 3:** Draft a practice improvement plan — based on the selected strategies, each practice drafts an improvement plan based on the SMART methodology. TOOL-kit includes step-by-step guidance for each strategy to support practices in writing the draft

**Step 4:** Implement the practice improvement plan – each practice implements the contents of the final improvement plan

During the trial, coordinating GPs received TOOL-kit and were able to choose which strategies they felt were most feasible and effective in their situation. We did not provide practices with criteria on the number of strategies or how TOOL-kit should be used in practice.

### Process indicators

The implementation of TOOL-kit components was evaluated using five process indicators based on the Saunders’ paradigm, a framework for evaluating programme implementation.^50^ This framework was selected because it is often used in evaluations of complex health interventions. The five process indicators were:

*Recruitment* refers to methods for including practices and participants, as well as the number of recruited practices and participants.

*Reach* is defined as the number of practice employees and patients receiving the intervention and its potential effect. The number of patients was estimated at baseline based on the number of GPs in each practice and their part-time factor times the number of patients of a standard Dutch practice (2095 patients).

*Fidelity* relates to the extent to which the steps of TOOL-kit were completed and to which the guidance of TOOL-kit was followed.

*Dose delivered* is defined by the number of strategies in the practice improvement plan, the extent to which the goals were compliant to SMART guidelines, and the extent in which these plans were of added value for the practice organisation.

*Dose received* is defined as the extent to which the targets included in the practice improvement plans were completed during the trial (short-term) and the impact of the intervention on practice organisation after trial conclusion (long-term).

### Barriers and facilitators for implementation

Based on the framework of Fleuren *et al*, we defined the following categories of determinants, which were related to: (1) TOOL-kit characteristics; (2) adopting persons (that is, practice staff and coordinating GPs); (3) general practice organisation; and (4) sociopolitical context^51^ (Supplementary Information S1). Impact of the COVID-19 pandemic on the determinants was addressed separately. We used the resulting 23 determinants in the qualitative analysis of the interviews.

### Data collection

**Figure 1.**
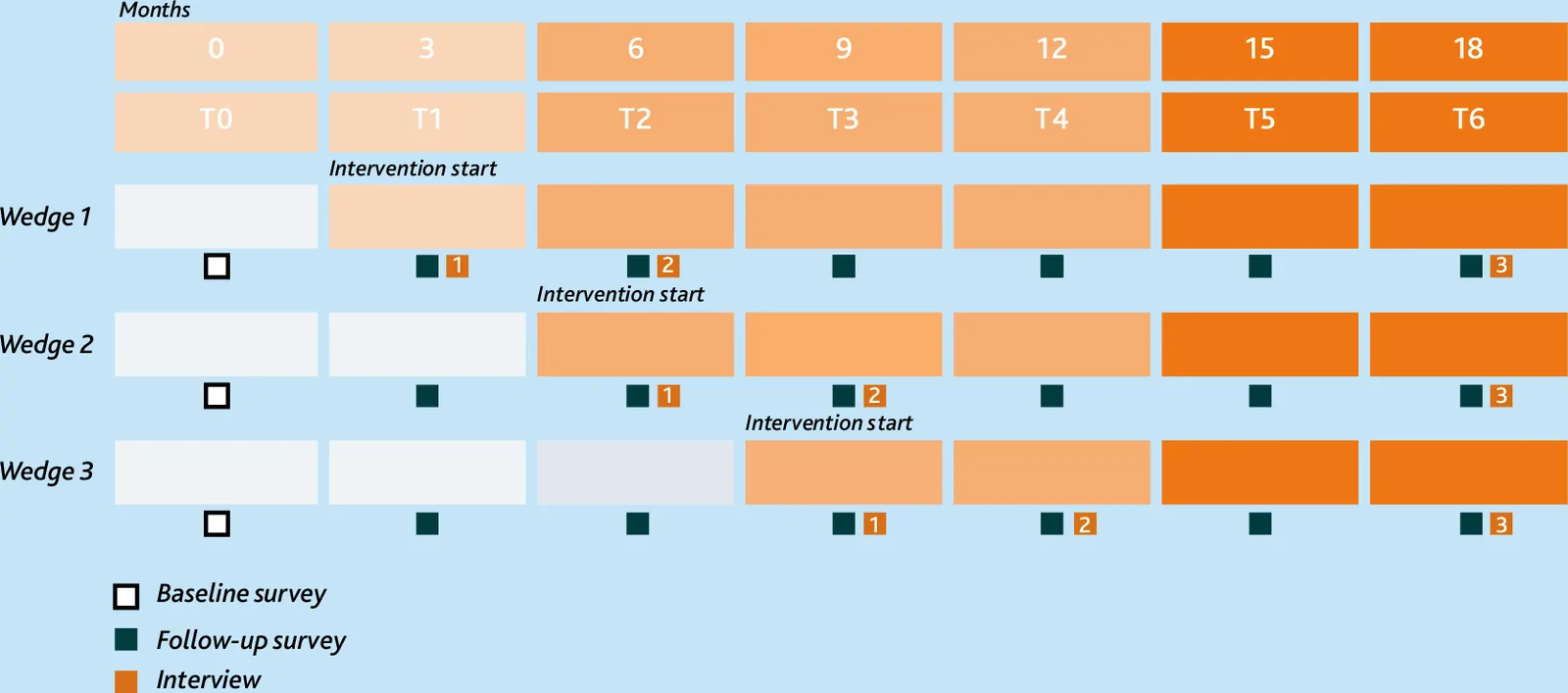
Data collection outline. Thirty-one practices were randomised in three wedges. After baseline measurement (**T0**), digital surveys (**T1–T6**) were disseminated, and interviews (**T1–T4, T6**) were held. T = timepoint.

Process evaluation was embedded in the data collection of the effectivity trial of the TOOL-kit from August 2020 to April 2022 (Figure 1). Data were collected using a tracking log, digital surveys, and interviews.

### Tracking log

Data on practice recruitment, inclusion and primary and secondary components’ rollout, were captured by the first author during the entire trial using Microsoft Excel.

### Digital surveys

The baseline survey (T0) collected data on participant characteristics. The follow-up surveys (T1–T6) evaluated the primary TOOL-kit components by sending coordinating GPs digital 3-monthly surveys. Coordinating GPs were asked to report on the progress and activities performed according to the plan and to express their level of satisfaction with the process.

After 24 months, we emailed coordinating GPs to ask whether they still used TOOL-kit and if they were actively promoting personal continuity in their organisation.

### Interviews

For evaluation of barriers and facilitators of the primary components, a total of three semi-structured interviews were held for each practice, resulting in a total of 93 interviews.

Interview one and two were conducted by telephone with the coordinating GPs individually. The first interview was scheduled 1 month after receiving TOOL-kit and focused on the user’s experience with the digital TOOL-kit interface and their first reaction to TOOL-kit. The second interview was scheduled 3 months after receiving TOOL-kit and explored the continued use of TOOL-kit in the practice and the preliminary results of its adoption into practice.

The third interview was held at the study conclusion and was set up as a group interview with coordinating GPs and other relevant practice staff. These interviews were conducted via video-conferencing using Microsoft Teams. The aim of this final interview session was to assess the results of the implementation process and investigate potential factors contributing to this result.

Interviews were conducted by the first author and a trained student assistant. Interviews took 15–30 min. All interviews were audio-recorded and transcribed verbatim by a third-party service. A conversation guide was used for all interviews.

### Analyses

Recruitment and reach were determined based on the tracking log using descriptive statistics (mean, percentages).

Data from the tracking log, surveys, and interviews were used to determine fidelity, dose delivered, and dose received. The data were evaluated using a tailored evaluation form to categorise and summarise the data (Supplementary Information S2). The first author reviewed the data and used the form to determine fidelity, dose delivered, and dose received using descriptive statistics (means, percentages). Dose delivered was categorised in four levels (very low, low, high, very high) based on the extent of adherence to the SMART guidance and the added value of TOOL-kit for the practice.

Barriers and facilitators were analysed with interview data using thematic analysis.^52^ The first author and a trained student assistant first familiarised themselves with the transcripts. The first one third of transcripts were analysed independently using codes based on the 23 determinants of the Fleuren framework (Supplementary Information S1) and open coding. The resulting codes were compared with the codes of the Fleuren determinants, discussed with the second author, and adjusted where appropriate, after which a preliminary categorisation of codes was established. The codes from this categorisation were then used by the first author to code the remaining interviews. The results of this process were evaluated and discussed with the second author to make the codes consistent with the determinants from the Fleuren paradigm. Barriers and facilitators were formulated from these definitive codes.

## Results

### Process indicators

#### Recruitment and reach

A total of 141 general practices were approached, 97 were eligible for participation and a total of 31 practices participated in the trial. Reasons for non-participation were non-response (*n* = 31), no capacity to carry out the intervention (*n* = 21), or no interest in the research topic (*n* = 14). The participating practices employed a total of 152 GPs, 172 practice assistants, and 116 practice nurses. An estimated 186 263 patients were registered at these practices at baseline. Coordinating GPs and practice characteristics are detailed in Supplementary Information S3. Other participant characteristics, including patient characteristics, are detailed in the main trial publication.^43^

#### Fidelity

Fidelity outcomes are summarised in Figure 2. All practices completed the first two steps of TOOL-kit, and 23/31 practices completed all four steps. Most (15/23) of these practices used TOOL-kit guidance to formulate the practice improvement plan in step 3.

**Figure 2.**
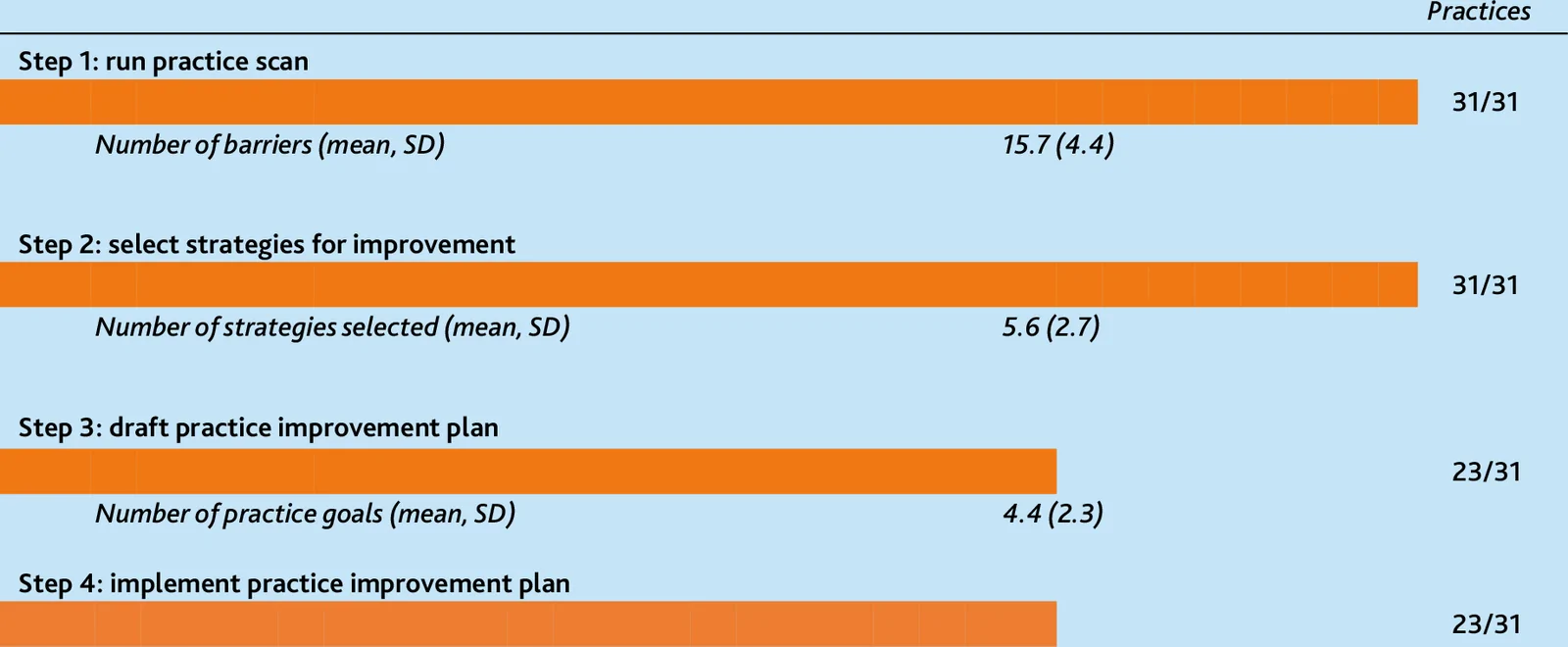
Fidelity of TOOL-kit use. SD = standard deviation.

#### Dose delivered (SMART compliance and added value for practice)

Some practices were described as having a very low dose delivered (*n* = 3). These practices did not implement TOOL-kit as their organisation already had a strong focus on personal continuity at study start and therefore perceived no added benefit from the intervention. Practices with a low dose delivered either only a partially drafted plan that did not adhere to SMART guidelines (*n* = 7) or used TOOL-kit solely to fine-tune their existing plans without drafting an additional SMART-compliant plan (*n* = 7). Practices with a high dose delivered (*n* = 13) successfully used TOOL-kit to develop a plan adhering to SMART guidelines, but did not report gaining new insights from TOOL-kit. Finally, one practice with a very high dose delivered formulated a SMART-compliant plan and reported gaining new insights from TOOL-kit.

#### Dose received (completion of plans and impact of the intervention after trial conclusion)

Practices achieved 55% (standard deviation [SD]: 25%) of the organisational goals set out in their practice improvement plan by study conclusion (average across all practices). At 24 months follow-up, 61% (19/31) of the practices reported still experiencing a positive effect from the intervention on their practice organisation in relation to personal continuity. This positive effect consisted of increased awareness of the importance of continuity (*n* = 6), to actively applying an improvement plan for continuity from TOOL-kit or otherwise (*n* = 8), or having set up a PDCA-cycle for personal continuity (*n* = 5). The other practices indicated they were not actively promoting personal continuity (*n* = 6) or did not respond (*n* = 6).

### Barriers and facilitators

The barriers and facilitators are summarised in Box 2. A complete overview of determinants is included in Supplementary Information S4. We elaborated on the most relevant barriers and facilitators in the paragraphs below.

**Box 2.** Summary of facilitators (F) and barriers (B)

**Determinants related to TOOL-kit characteristics**	
**Relative advantage**	TOOL-kit (F) is or (B) is not perceived as advantageous
**Appeal and user-friendliness**	TOOL-kit (F) is or (B) is not user-friendly, feasible, and acceptable
**Compatibility**	TOOL-kit is (F) relevant or (B) irrelevant to practice work procedures TOOL-kit (F) aligns or (B) does not align with practice organisation structure
**Determinants related to the adopting person(s**)	
**Task orientation**	Practice (F) strives or (B) does not strive for uniform working method among GPs
	Practice improvement plans are (F) systematically evaluated, for example, through random sampling or (B) are evaluated based on ‘gut instinct’ and on an ad hoc basis
	GPs (F) perceive themselves as academic doctors and have affinity with making improvement plans or (B) perceive themselves as practical doctors and have no affinity with making improvement plans
**Work-related stress**	Team (F) experiences acceptable workload and perceives to be in ‘calm waters’ or (B) feels overwhelmed and perceives to be in ‘survival mode’
**Skills and knowledge**	Practice assistants have (F) sufficient or (B) insufficient knowledge of importance of personal continuity
	Coordinating GP (F) has or (B) has no insight into practice organisation, for example, (F) by being the practice owner
**Expected patient reaction**	Coordinating GP expects patients to (F) appreciate or (B) not appreciate the results of TOOL-kit
**Ownership**	Coordinating GP takes (F) sufficient or (B) insufficient responsibility for TOOL-kit
	Coordinating GP (F) believes or (B) does not believe personal continuity is an important and urgent issue in their practice
**Self-efficacy**	Coordinating GP (F) has or (B) has no confidence that they can make TOOL-kit work in their practice
**Support from practice staff**	The team (F) supports or (B) does not support TOOL-kit use and implementation
**Determinants related to general practice organisation**	
**Decision-making process and organisational size**	In larger practices, (F) there is or (B) is no broad expertise and support for implementing interventions Collaboration is important to create support but is also challenging in a larger practice organisation
**Reinforcement by management**	Management (F) ensures or (B) does not ensure continuous quality improvement
	Management (F) supports or (B) does not support striving for quality improvement
**Structure and formalisation of quality improvement**	Practice (F) has or (B) has no designated quality officer(s)
	Practice has (F) or (B) has no quality improvement structure, that is, regular team meetings and a certain hierarchy
**Staff capacity**	Staff are (F) available or (B) not available to support TOOL-kit execution
**Staff turnover**	Practice has (F) a full and stable team or (B) staff shortage, high turnover, and many locums
**Determinants related to the sociopolitical context**	
**Patient awareness of continuity benefits**	Personal continuity (F) is or (B) is not important for patients, according to the coordinating GP
**Rules, regulations, and legislation**	Parts of TOOL-kit are perceived as (F) fitting or (B) not fitting Dutch legislation for patient right to freely choose their GPs and European privacy legislation
	Reimbursement by health insurance companies is (F) sufficient or (B) insufficient

#### Determinants related to TOOL-kit characteristics

Overall, coordinating GPs felt TOOL-kit was easy to use, feasible, and provided adequate guidance for the development of improvement plans:

*‘Improving personal continuity is a custom job and TOOL-kit succeeded in providing a proper base for that: what is patient-centred care, what topics are relevant in that context, and what first steps could you make as a practice. I think there are many practices who could really benefit from TOOL-kit.’* (Coordinating GP, practice #7)

Although TOOL-kit was reported to be compatible with larger practice organisations, coordinating GPs of smaller practices felt the intervention was not always relevant to them:

*‘We’re a really small practice. We only have 2 GPs and one locum GP and a small team of practice assistants. Likewise, we feel we already provide proper continuity of care and we felt many interventions from TOOL-kit were not useful to us.’* (Coordinating GP, practice #12)

#### Determinants related to the adopting person(s)

The characteristics of the coordinating GP influenced TOOL-kit implementation. Some coordinating GPs felt TOOL-kit fitted their task orientation and capabilities well:

*‘I’m one of the practice-owners so I know all procedures of the practice organisation, which made it easy to use TOOL-kit.’* (Coordinating GP, practice #28)

Other coordinating GPs experienced more difficulties using TOOL-kit:

*‘I'm not a researcher, I'm a doctor. I find it quite complex to organise TOOL-kit properly and keep a good grasp on it: what is your goal, how do you keep it measurable.’ *(Coordinating GP, practice #17)

Support of the team was considered necessary for TOOL-kit to succeed. Support of general practice staff was often attained:

*‘I do think we have more collaboration with the whole team* […] *because this project is more organisational, I do feel we are much more in it together.’* (GP, practice #9)

However, coordinating GPs did not always succeed in gaining this support:

*‘Everyone is just busy putting out fires everywhere. Of course, I try to consider what things I can do alone and what things can be delegated. But when I try to delegate, the task often comes back to me like a boomerang.’* (Coordinating GP, practice #25)

In many practices, a high amount of work stress was experienced in combination with high staff-turnover and low capacity. Coordinating GPs of these practices often described their situation as ‘*unstable*’ and being in *‘survival mode*’:

*‘We’ve been too busy putting out fires; a huge staff shortage for both practice assistants and GPs* […] *The staff does not feel any room to do anything extra, even if it could be something fun, it’s a complete no-go at the moment.’* (Coordinating GP, practice #13)

Other practices experienced stability and adequate staffing and felt this facilitated TOOL-kit implementation.

#### Determinants related to general practice organisation

Coordinating GPs of larger general practices perceived having a larger scale setting to be beneficial as there is more diversity of expertise and more administrative support available compared with smaller practices. However, they also felt that the larger setting increased the need for a more elaborate and structured decision-making process and designating staff for quality improvement projects:

*‘I think it’s truly a part of our core business to keep evolving rather than seeing our basic primary care as the foundation, with additional activities as extras. We truly see continuous improvement as a pillar of our practice. We have our basic care, which continues on a day-to-day basis, but we're constantly in a long-term improvement cycle.’* (Coordinating GP, practice #7)

However, other larger-scale practices indicated they did not have a structured decision-making process and were not able to designate staff to the TOOL-kit project. These practices tended not to prioritise TOOL-kit when the workload rose.

#### Determinants related to sociopolitical context

GPs often struggled with patients choosing accessibility of GP care over continuity of care:

*‘People want to be helped more quickly, and sometimes it doesn't even matter if it’s with a different general practitioner, which can lead to enormous fragmentation. That can be acceptable for minor issues, but you have to keep in mind that through those small things, the patient may also be sending a message. So, you still need to maintain an overview of the whole situation.’* (Coordinating GP, practice #21)

Most coordinating GPs encountered no legal obstructions in adopting TOOL-kit, although some practices did not send a newsletter to patients because they felt this did not comply with European data protection laws.

#### Impact of the COVID-19 pandemic on determinants

The COVID-19 pandemic was considered a barrier for implementation in most practices. The pandemic exacerbated work stress owing to direct care for patients with COVID-19 and administering vaccines, in combination with decreased staff capacity owing to COVID-19 infections or quarantine. Many practices felt it was difficult to apply TOOL-kit in this healthcare situation:

*‘The difficulty of this year is that it’s hardly representative. Because, after the first evaluation of TOOL-kit, a lot has changed and there is a lot of chaos in general and especially in the hospital. It is quite hard to maintain continuity of care at the moment, let’s put it that way. It’s not always a priority for us now.’* (Coordinating GP, practice #19)

## Discussion

### Summary

This study aimed to evaluate the implementation of an intervention to improve personal continuity for older (aged ≥65 years) patients (TOOL-kit) and to discover what factors influenced its application in general practice. It was found that while the fidelity (adherence to protocol), was high, the dose delivered (extent of improvement plans) was (very) low with 17/31 practices drafting an improvement plan. Dose received (goal achievement, sustained organisational change) was considered moderate with an average goal achievement rate of 55% at study conclusion. In addition, TOOL-kit did demonstrate long-term impact by enhancing work procedures to optimise personal continuity and/or inducing awareness of the importance of personal continuity in 19/31 practices. The TOOL-kit was perceived as beneficial and user-friendly while its application was perceived to be hindered by work stress and staff shortages, exacerbated by the COVID-19 pandemic. Practices indicated that having a structured decision-making process, designated staff, a full and stable team, and an organisational culture that promotes quality improvement were supportive factors for TOOL-kit implementation.

### Strengths and limitations

The process evaluation combined quantitative and qualitative data to perform a comprehensive analysis of TOOL-kit implementation. The use of surveys tailored to the practices, combined with interviews, allowed for the collection of an in-depth narrative of the application of TOOL-kit into practice and quality improvement in general practice.

All data collection in this study relied on self-reporting by the coordinating GPs. While this is common in process evaluations, there is a chance of self-serving bias. This makes it more difficult to distinguish what was actually done and what the participants reported having done. In addition, most of the data collection of the process evaluation was done with the coordinating GPs and not with other practice staff or patients. This approach was chosen because previous research indicated that patients are often not familiar with the details of practice organisation.^42^ Other practice staff were invited for the final group interview. However, this may not have ensured a comprehensive involvement as views divergent from the majority are not always expressed in a group setting. The inclusion of direct observations and individual collection of process data among other practice staff might have resulted in a more complete perspective.

Many practices were already active with initiatives to maintain or improve personal continuity before the study started.^53^ In addition, other external programmes may have influenced TOOL-kit’s implementation changes. These initiatives were not mapped out at the start of the study. As such, it can be difficult to discern the effect of TOOL-kit from already running initiatives. For example, several practices succeeded in implementing 15-min consultations during the trial period, which was included in the TOOL-kit, but also strongly facilitated by the nationally ongoing More Time for the Patient initiative.^54^ However, the extent to which practice improvement plans were actively developed was described and used in this manuscript and in the main trial publication.^43^ Therefore the process evaluation sufficiently describes this phenomenon.

Practices often paused, delayed, or sometimes stopped applying TOOL-kit in their practice owing to the consequences of the COVID-19 pandemic (that is, staff shortages, quarantine, vaccination campaigns, increased COVID-19 patient care load). In addition, owing to the COVID-19 pandemic, follow-up duration was reduced from 24 months to 18 months. Therefore, the level of implementation might have been higher if COVID-19 had not come to pass.

### Comparison with existing literature

Although interventions for improving personal continuity are available, they are often not formally evaluated and often have a weak evidence base.^44^ Interventional studies with structured process evaluations are reported rarely.

An Australian multi-component intervention to improve continuity of care showed limited improvement in continuity and no changes in patients’ health outcomes despite achieving a reasonable level of implementation.^55^ A subsequent qualitative study evaluating the intervention found that practice staff perceived the intervention as beneficial and supported the intervention.^56^ However, practice staff felt that the intervention contents had already been part of usual care of the participating practices, which may explain the lack of significant changes during the trial.

Another randomised control trial (RCT) on an Australian multi-component intervention to improve continuity of care, consisting of patient enrolment and financial initiatives, also found no effect.^57^

In a mixed-methods evaluation, the authors found that insufficient support of financial incentives and pre-existing organisation routines regarding personal continuity of care between GPs and their patients may explain the lack of measurable effect.^58^ Additionally, the authors described the intervention did lead to an increased focus and small adjustments in routines towards personal continuity.

These findings align with the results of this study, as only one practice indicated that TOOL-kit provided novel insights to them. The absence of measurable effects, combined with participants’ positive perceptions of the intervention and their sense that it contributed to changes in work procedures aimed at improving continuity of care, also represents a shared result.

In the UK, The Health Foundation’s Increasing Continuity of Care in General Practice programme supported five large GP organisations in implementing a range of continuity-focused quality improvement interventions.^59^ These interventions focused on staff and patient engagement, patient allocation, workflow redesign, and the measurement of continuity. The mixed-methods evaluation found that such interventions can improve quality and efficiency, with the most sustainable changes combining new processes with a broader cultural shift towards continuity.

This study confirms these findings, as it was found that practice organisation and an organisational culture prioritising quality improvement and personal continuity is important when implementing interventions to optimise personal continuity. The approach in this study differs from the more pragmatic approach in the Increasing Continuity of Care in General Practice programme as TOOL-kit was based on a broader and more substantiated inventory of strategies developed before the study started.

In this study we found that organisational culture may influence implementation of TOOL-kit. Organisational culture includes shared beliefs, norms and values, and routines among staff within the same organisation.^60,61^ The importance of organisational culture on the implementation of healthcare innovations is well known.^62–64^ However, it is not often recognised as a determinant in research on barriers and facilitators in general practice.^65^^,^^66^ TOOL-kit does not contain strategies addressing organisational culture and no effective strategies have been identified in the literature.^60^

### Implications for research and practice

This study demonstrates that successful implementation of a complex intervention is not guaranteed even when it is perceived as beneficial and acceptable. The findings suggest that the organisational structure and culture of general practices considerably influence the successful implementation of TOOL-kit.

To increase the likelihood of successful implementation of new innovations in general practice, general practice management should invest in robust quality improvement frameworks, including clear roles and responsibilities for the employees, structured problem identification followed by a solution development processes, effective implementation strategies, and regular evaluation of outcomes. Moreover, quality improvement should be prioritised within the practice, rather than being relegated to a secondary role amid the demands of patient care. Regional practice networks can serve as facilitators in implementing this process.

Future research on the implementation of innovations in general practice should assess organisational structure and culture in relation to quality improvement and incorporate the strategies mentioned above. It should also alert participating practices that a focused approach to transforming organisational structure and culture is needed to support quality improvement. More research is needed to address organisational culture as a determinant of implementation to develop approaches to improve continuity in general practice.

The findings indicate that the general shortage of staff, high workload, and high staff turnover in general practices are conditions negatively influencing implementation that are harder to influence. Individual general practices are not equipped to tackle these systemic issues. However, a focused approach to tackle these issues also seems to be lacking on the sociopolitical level. A societal discussion is required to address these broader problems. This should involve a dialogue between GPs, patients or their representatives, and policymakers on a national level, addressing the increased administrative demands, increased tasks of GPs, and finding a balance between timely access, continuity of care and feasibility.

TOOL-kit was evaluated in a pragmatic setting with limited guidance and support for practices. This resulted in an intervention that was feasible and compatible but might have been too aligned with strategies already part of usual general practice organisation. Testing TOOL-kit in a setting with more financial and organisational support may provide insight in the benefits of longitudinal investment in primary care in the longer term.

In conclusion, despite implementation challenges, TOOL-kit was able to make a long-term impact at practice level by increasing awareness of the importance of personal continuity and enhancing work procedures related to personal continuity. The findings demonstrate the need for certain conditions to be met before implementation can be successful. General practices could improve implementation chances by establishing a clear structure on decision making related to quality improvement, designating staff to these projects, and being more vocal on the importance of continuity of care and quality policy within practice. A national dialogue between GPs, patients, and policymakers is required to address the broader problems of shortage of staff, high workload, and high staff turnover.

## Data

The data are not publicly available due to the qualitative nature of the data, which could comprise the privacy of reasearch participants. Request for dataset can be sent to the corresponding author for individual assesment of feasability.
